# “Bringing the outside world in”: Enriching social connection through health student placements in a teaching aged care facility

**DOI:** 10.1111/hex.12561

**Published:** 2017-04-11

**Authors:** Michael J. Annear, Kate‐Ellen J. Elliott, Laura T. Tierney, Emma J. Lea, Andrew Robinson

**Affiliations:** ^1^ Wicking Dementia Research and Education Centre (WDREC) Faculty of Health University of Tasmania Tasmania Australia; ^2^ School of Health Sciences Faculty of Health University of Tasmania Tasmania Australia

**Keywords:** aged care, quality of life, student placements

## Abstract

**Background:**

Older adults living in residential aged care facilities (RACFs) often experience limited opportunities for social connection despite close proximity to peers, which has implications for mental health and quality of life (QoL). The introduction of large‐scale undergraduate health student placements in RACFs may enhance opportunities for meaningful engagement through social connection, although this remains unexplored.

**Objective:**

This research explores whether interpersonal encounters between health students and RACF residents influence residents’ opportunities for social connection and QoL.

**Methods:**

A mixed methods design was employed which included questionnaire data from residents, and qualitative interview data from residents, family members and RACF staff. Data were collected during and after student placements to allow for an in‐depth exploration of residents, family members and staff perspectives.

**Results:**

Forty‐three participants (28 residents, 10 staff and five family members) were recruited during 2014. Overall, many residents had clinical levels of depression, mild cognitive impairment and multiple morbidities, however reported moderate‐to‐good QoL. Thematic analysis was undertaken on interview transcripts, and three themes emerged: (i) social isolation and loneliness fostered by residents’ age‐related conditions, (ii) students expand socially supportive connections beyond the RACF and (iii) meaning making by sharing health experiences, which was found to help renegotiate older adults’ pervasive narrative of vulnerability.

**Conclusion:**

Supported and structured health student placements in RACFs enable older adults to participate in meaningful encounters with younger people. These encounters focus on sharing health experiences and address long‐standing issues of isolation and loneliness by providing opportunities for social connection.

## 1 INTRODUCTION

Interpersonal relationships are central to human functioning. The influence of social connection on well‐being is particularly prominent in older adulthood. A meta‐analysis of findings from longitudinal studies of older adults demonstrated that the risk of mortality halved when participants had strong social relationships.^1^ Around the globe, most older adults live in their own homes in familiar communities.^2^ However, a significant proportion of very old people are unable to maintain independent living due to progressive health decrements, which often necessitates a move into a residential aged care facility (RACF). Arguably, living alongside other older adults in a RACF, often sharing a hallway or dining area with several peers, should provide opportunities for meaningful social connection. Several reports, however, indicate that these health expectations are not met. Loneliness is a potentially significant yet relatively unrecognized issue in the context of RACFs and is defined as the perceived gap between actual social relations and desired ones.^3^, ^4^, ^5^, ^6^ In such settings, residents often lack the necessary stimulation and interaction to exercise agency in a manner that is meaningful and fulfilling.^7^ This is problematic because the effects of loneliness are detrimental for older adults’ health and well‐being. Without intervention, this problem is likely to persist and be exacerbated as population ageing accelerates worldwide.^8^, ^9^

Social connection in RACFs is a challenge for older adults when chronic conditions limit mobility and access to social groups.^6^ Access to meaningful activities for residents, which may include opportunities for social connection, is also restricted, and traditionally, the culture in RACFs has been known to result in limited stimulation.^7^ Evidence suggests there is a lack of human resources within RACFs to facilitate meaningful engagement and interaction among residents.^7^ This may be due to funding structures that emphasize physical needs over psychosocial care,^10^ as well as high staff turnover and low recruitment rates.^11^ Despite the evidence that supportive social connections in older adulthood can act as a resource to help buffer against stressors^12^ and enhance capacity for coping with chronic conditions through shared coping styles,^13^ the need for social connection apparently remains unmet in this setting.^6^, ^14^, ^15^

Symbolic interactionism is a theory that provides a perspective for understanding the benefits of increased socially supportive connections, which can be applied to older adulthood. This approach describes how meaning is generated subjectively through social interaction and role‐taking that occurs when two or more individuals come together in a reciprocal transaction.^16^, ^17^ Older adults who experience low mobility and have high care needs may have fewer opportunities for activities that promote the processes (e.g, role taking) that characterize symbolic interactionism. Herbert Blumer's^18^ original conception of symbolic interactionism is deeply rooted in the understanding that social interaction is critical to the formation of meaning and identity across the life course. The overarching tenets of symbolic interactionism include, (i) that people act toward things, including each other, on the basis of the meanings they have for them; (ii) that these meanings are derived through social interaction with others; and (iii) that these meanings are managed and transformed through an interpretive process that people use to make sense of and handle the objects that constitute their social worlds.^18^ According to this theory, the ability to generate meaning at all life stages is dependent upon the interplay of individual agency, external stimulation and interaction between individuals.^19^ It is the nature of social interaction and what Blumer referred to as social worlds^18^ that may change when a person moves from their home in the community into a RACF. Intervening via external stimulation in the environment may be one way to maximize opportunities for social connection for older adults. For example, teaching aged care facilities,^20^ where students undertake practical training in health disciplines, may provide new opportunities for social exchanges with residents. Such social exchanges may have the effect of prompting memory (as a form of reminiscence), consolidating personal identity and creating the conditions for increasing the level of meaning in one's life. Independent of student placement programmes, other psychosocial behavioural interventions, including reminiscence therapy, with aged care residents who have dementia, have proved to be an efficacious prompt for the recall of memories and reconnection with early life identity.^21^, ^22^

Large‐scale student placements in RACFs have the potential to offer new opportunities for social interaction and possible social connection among older residents, yet the effects of such programmes have rarely been researched. Students’ learning activities may provide a unique opportunity for older adults to become active participants in reciprocal social interactions.^23^ RACFs are underutilized for health student placements compared to other settings, such as hospitals or general practice.^24^ Most Australian undergraduate health student placements (including medical, nursing and allied health disciplines) are located in hospitals, representing 63% of total placement hours.^24^ In contrast, only 3.5% of these clinical placement hours are undertaken in RACFs.^24^ Despite the low frequency of health student placements undertaken in RACFs, findings in this emerging field of research have reported benefits for older recipients of student‐delivered care.

Preliminary mixed methods studies suggest that short‐term relationships between undergraduate health students and older adults living in RACFs may contribute to improved health and quality of life (QoL),^23^, ^25^ particularly via student‐resident social interaction. Resident and family member perceptions indicated improvements in capacity for care, facility atmosphere and opportunities for social interaction at two teaching aged care facility sites. In addition, health student placements provided a context for residents to express altruism by being central to learning activities, such as health assessments.^23^ In further support of these preliminary findings, social interaction has previously been found to be a key influence on QoL for older adults living with dementia in Australian RACFs.^26^ The capacity of students to provide psychosocial support for residents may be highly significant due to the potential for isolation and loneliness within RACFs, although this topic has received scant attention in the international literature. This study aims to extend the preliminary findings reported above^23^ by exploring the interpersonal encounters between health students and RACF residents and examining how these may influence opportunities for meaningful social connection and QoL for older adults.

## 2 METHODS

### 2.1 Setting

The study setting was a 140‐bed RACF in Tasmania, which has hosted large‐scale interprofessional health student placements since 2011 as part of the Wicking Teaching Aged Care Facility (TACF) Program.^20^, ^27^ Since publication of the pilot findings,^23^ the numbers of students who attend placements at the TACF have increased as the capacity for supervision has improved and a university learning centre has been established within the grounds of the RACF. During this study (semesters one and two, 2014), 95 students from three disciplines (n=45 medicine, n=30 nursing, n=20 paramedicine) completed clinical placements. Placement lengths ranged from one week (medical, paramedic) to four weeks (nursing). Students interacted with volunteer residents for up to 16 hours each week and completed comprehensive health assessments with interdisciplinary student peers as part of an interprofessional learning activity.^28^, ^29^

### 2.2 Design

A mixed methods design was employed to gather data during and after student placements. The mixed methods design was conceived as QUAL‐QUANT, with qualitative data used to provide the main explanatory framework around contextual quantitative information concerning residents’ physical and mental health status. Data were collected in parallel, and the study was considered as an exploratory investigation due to limited evaluation of the outcomes of TACF in Australia or similarly developed countries. No experimental condition was used in this study, although the research focussed on the potential effects of an on‐going intervention—the Wicking TACF student placement programme.^20^ Residents, family members and aged care staff were recruited to provide their perspectives on the health students, while placements were underway and two weeks after their completion. This approach permitted the collection of rich, in‐depth data as well as saturation for the purposes of qualitative thematic analysis. Data from three cohorts (residents, family members and RACF staff) were used to provide verification of perceptions about the efficacy and outcomes of the student placement programme, and two modes of data collection were employed to enhance the richness of the data.^30^ Questionnaire data were collected on residents’ levels of cognitive impairment, depression and QoL. Semi‐structured interviews were conducted with residents, family members and staff on the impact of students’ presence in the RACF on residents’ well‐being and QoL. This study received approval from an institutional human research ethics committee (Ref. No. H12771).

### 2.3 Sample

Older adult residents in the RACF, their relatives (listed as contacts in facility records) and staff who provide daily care to residents were invited to participate in the research between May and November 2014. Inclusion criteria for residents were as follows: (i) a psychogeriatric assessment scale cognitive subscale (PAS‐CS) score of 9 or lower (indicating mild cognitive impairment with capacity to provide informed consent^31^) and (ii) process consent (subjective evaluation of ability to participate) undertaken by a facility staff member on both day of consent and day of research participation.^32^ Residents had diverse exposures to students, which included contact for the purposes of health and social assessment undertaken at the time of this study. The on‐going nature of the placement programme, which began in 2011, also meant that many residents had prior contact with other students associated with past placements. Residents’ family members were invited to participate via a letter from the RACF. Family members were well placed to describe changes that they had observed in their relative as a consequence of interactions with students. Staff members who provide regular care for participating residents were recruited by a senior nurse and referred to the first author who invited their participation. Staff members were able to comment on the nature of student interactions (in terms of care provision, social interaction or supervised treatment and assessment) and resident responses to and reflections upon the student cohort. All respondents provided informed, written consent for participation consistent with the human ethics approval.

### 2.4 Measures

Qualitative and quantitative data were collected during and after two, four‐week student placements (that included all student groups in different combinations). Three quantitative scales were used to provide initial explanatory data that helped to define and contextualize the cohort under investigation and determine their level of vulnerability. General demographic data were also recorded from patient records. The validated 15‐item, Long‐term Care version of the Quality of Life survey (QoL‐LTC)^33^ was administered to residents, family members and facility staff. Participants rated their health‐related QoL on a four‐point Likert scale (1 = poor, 2 = fair, 3 = good, 4 = excellent). As an example, one item from the scale was worded as follows, “How do you feel about your physical health? Would you say it is poor, fair, good, or excellent?” Possible scores range from 15 to 60, and a higher score represents better QoL. Residents provided permission to gather information from their facility files from routine general and mental health assessments. This included measures of cognitive impairment (PAS‐CS, as stated above), depressive symptoms and chronic health conditions.^31^, ^34^ Depressive symptoms were measured by the 19‐item Cornell Scale for Depression^34^ on a three‐point Likert scale (0=absent, 1=mild to intermittent, 2=severe), with higher scores indicative of greater frequency of depressive symptoms. Semi‐structured interview questions addressed resident health status, QoL, quality of care and interactions with health students. Examples included, “describe your interactions with students this week” (resident); “how, if at all, has your relative's QoL or emotional state changed while the students have been on placement” (family member); and “tell me about possible benefits for residents who have contact with the students” (staff). Questionnaires and interviews were administered concurrently and undertaken face‐to‐face by a gerontologist (MA) and a public health researcher (LT).

### 2.5 Analysis

Questionnaire data were analysed using SPSS version 20.^35^ Using SPSS, data were cleaned, normal distributions were verified and descriptive statistics were used to report scale scores, including means, standard deviations and value ranges. Total scores on measures were tallied as per author instructions, and group means were calculated. Qualitative data were analysed following a two‐step coding procedure using NVIVO software.^36^ Interview transcripts were scanned for categorical statements (initial codes) relating to student‐resident interactions and outcomes. Subsequently, analytical (focussed) coding was used to identify respondent statements that best reflected underlying or latent themes. A thematic description was then developed for each collection of analytic codes. The first author (MA) coded all transcribed interview data. The third author (LT) then coded a random selection of 25% of the transcripts, and levels of agreement were calculated using a coding comparison query.

## 3 RESULTS

Forty‐three respondents participated in this research, including RACF residents, family members and staff (nurses and care workers). Of 34 residents invited to participate, 28 provided written consent (82% response rate) and six did not take part. Reasons for nonparticipation included acute illness (n=1), death prior to participation (n=1), refusal without explanation (n=3) and failure to meet process consent criteria (n=1). Within three months of data collection, six residents (21% of the sample) were deceased. All 10 staff (nurses and care workers) invited to participate provided consent for involvement in the research (100% response rate). Relatives of residents had the lowest recruitment rate with only 5 of 15 who were contacted via letter providing written consent (33% response rate). Reasons for nonparticipation were not provided.

Residents who participated were mostly women (mean age=86; SD=10) with no close partner relationship (75% were women and 82% were widowed, divorced or never married). Primary reasons for RACF admission were inability to cope at home (61%) and lack of caregiver capacity (18%). The most prevalent health conditions among resident respondents included probable depression (54%), dementia and short‐term memory loss (27%), cardiovascular disease (26%) and sensory impairments (11%). Residents reported their QoL as moderate‐good^37^ (see Table 1).

**Table 1:** Volunteer resident characteristics (n=28)

Demographic characteristic	Mean	Standard deviation	Range
Length of TACF residence (months)	40.00	37.13	10‐175
Psychogeriatric assessment scale‐cognitive subscale total score	4.26^a^	3.10	0‐9
Cornell depression scale (CDS) total score	12.07^b^	5.64	0‐23
Quality of life—long‐term care total score	41.02	6.89	27‐53
Total number of chronic health conditions per resident	2.93	1.24	1‐6

a A score of 4 is indicative of probable mild cognitive impairment, and a score above 9 is indicative of probable major cognitive impairment (such as dementia).^31^

b A score of 12 is indicative of probable depression, with 54% of respondents scoring 12 or greater.^34^

### 3.1 The experience of student interactions in aged care

The primary qualitative data analysis identified three themes through which student placements potentially supported resident QoL. All themes address the potential psychosocial impacts of the placements. Themes included the following: (i) social isolation and loneliness compounded by residents’ age‐related chronic health conditions, (ii) students expand social connection beyond the RACF and (iii) meaning making by sharing health experiences. Secondary coding analysis demonstrated the high levels of agreement between the two researchers who conducted the analysis (inter‐rater reliability; κ=0.96).

### 3.2 Theme 1: social isolation and loneliness fostered by residents’ age‐related conditions

Many residents felt isolated and lonely despite their close proximity to other residents and staff. While there are opportunities for older adults to be socially active (communal meal times, leisure and lifestyle classes, facility events and community excursions), significant frailty, periods of acute illness or exacerbation of chronic conditions often restricted residents to the immediate surrounds of their room or apartment. In these circumstances, residents described their only social interactions in terms of fleeting daily visits from carers, cleaners or nurses, and generally infrequent visits from family members. The experience of social disconnection from other residents and psychological impact of a physical health condition was recounted by a resident:

I don't interact very much with the other [residents] because of my hearing and my sight. I stay here [in my room] most of the time…I have my meal here because I go to the dining room and you sit around a table and everybody is speaking very quietly and you can't hear (OP21_Resident).

Residents reported frustration because of their perceptions of, not only their own, but others’ sensory impairments that limited capacity for meaningful engagement. One noted, *“…I keep getting asked questions* [by other residents]*, ‘how long have you been here, or have you got children?’ I might get asked that eight times in a month…I just answer and grin and bear it”* (OP20_Resident). Limited social engagement was also highlighted by residents’ sense of isolation, as one recounted: *“I just sit here all day. My family are at work. They can't come in during the day…you've got to entertain yourself”* (OP42_Resident). Family members echoed such comments. One stated what this meant for her father, saying, *“Because of his current* [poor] *health,* [my father] *is basically stuck in his room…I wouldn't call this a very good quality of life”* (OP31_Family member). Health issues overlaying complex multiple morbidities, which limited residents’ willingness and capacity to engage, compounded these kinds of problems. One family member commented about her mother who, at the time, suffered from a heart condition and a virus, noting that she had, *“… been missing out on her regular art sessions and not going to have her meals in the dining room. She's been eating in her own room not wanting to infect anybody else, but also just because she's weak”* (OP32_Family member). A staff member also highlighted how residents’ isolation could increase alongside progressive functional decline. She commented that for most residents *“…life is changing, there is no recovery, it is going to just keep going downhill,* [over time] *the isolation is actually going to become more of an issue ….”* (OP28_Staff). These qualitative data suggest that for many residents both poor quality of social engagement and a progressively declining capacity to engage contribute to a sense of isolation.

Difficulties in staff facilitating meaningful engagement among residents compound the problems described above, because as one staff member noted, *“…carers* [staff] *can only give so much* [interpersonal attention] *because there are so many residents requiring attention”* (OP39_Staff). The staff narrative indicates that time and human resource constraints, which are characteristic of aged care environments, limit their capacity to facilitate social engagement among residents. While residents described loneliness and isolation as a function of a lack of suitable and articulate companions, family members ascribed this to the progression of degenerative health conditions or bouts of acute illness or injury. Staff members saw both sides of this issue, noting residents’ changing life circumstances as a barrier to social engagement and their own lack of time to invest in supporting meaningful interaction.

### 3.3 Theme 2: students expand socially supportive connections beyond the RACF

Large‐scale health student placements provided an opportunity for increasing residents’ social contact when they were feeling isolated and lonely. As part of TACF placements, students participated in daily care and undertook comprehensive resident health and medical assessments. Residents who encountered the students spoke of their appreciation at the opportunity to spend time with younger adults who they perceived as showing concern for their physical and emotional well‐being. In particular, residents mentioned that students brought the *“outside world”* into the facility. For example, one resident commented, *“…it's nice to hear their* [students’] *stories. It's interesting to hear where some of them come from, the different places they were brought up…it brings the outside world back in for a few minutes”* (OP24_Resident). Similarly, another resident noted, *“The students brought you more of the outside world. I was interested to find out from them how they were going, what they were studying, and what stage they were at”* (OP07_Resident). Residents also recounted experiences of connection with students over the course of their placement. For example, referring to a nursing student who provided care over four weeks, one resident stated: *“We got on really well. She* [the student] *came in and sat with me for about an hour at the end* [of the placement], *which was lovely.”* (OP40_Resident). Residents perceived student interaction to be meaningful with a high degree of reciprocity. The aforementioned resident reported that the student, *“…brought me in some flowers and a lovely little card… wrote a poem for me… that thanked me for allowing her into my life”* (OP40_Resident). The impact of this relationship was evident to this man's family, with his adult daughter noting: *“Dad always talks to me about a medical student he's had…we were blown away by the fact that she came back a couple a days later and she'd written that beautiful poem for him. That just was such a beautiful interaction…* [it] *has just been very good for him*” (OP50_Family member).

On any RACF shift during the placement periods, there could be up to 20 medical, nursing and paramedic students within the RACF. In many instances, their presence improved both perceived quality and quantity of social interaction. A staff member respondent noted the impact of the large‐scale placements on residents’ level of social engagement. She contended, “*When the students are here the residents get a lot more time*.*”* Clarifying this, she said, *“They* [the resident] *might have someone in the room with them for an hour whereas we're struggling to give them 25 minutes”* (OP25_Staff). The capacity for students to spend quality time with residents was consistently reported during staff interviews, with comments such as *“…*[the residents] *probably had 30% extra interaction time…*[it's] *new beautiful quality time which eases the emotional pain of being in an aged care environment*…[because care staff] *do lack time”* (OP28_Staff), and *“When the students come here we're able to give that little bit extra care* [to residents]*”* (OP39_Staff). Staff, family members and residents acknowledged that student placements increased both the quality and quantity of social support, particularly emotional support, which is an important aspect of QoL in the RACF. This was important for many older adults who were confined to their rooms by illness or frustrated by a lack of meaningful social engagement with staff, family and co‐residents.

### 3.4 Theme 3: meaning making by sharing health experiences

Residents also valued the student placements as they provided a sense of greater meaning in their lives, fostered healthy adjustment to the aged care setting and gave something back to a future generation of health professionals. Some residents had a history as health professionals and educators, which they reconnected with when they interacted with medical, nursing and paramedic students. For these residents, the process of teaching students helped them to reclaim an important element of their personal identity. Accordingly, the data indicated that a key motivator of engagement was residents’ desire to contribute to students’ education. As one resident noted *“…we have to give something back”* (OP07_Resident).

It was evident that residents wanted to help students better understand the issues associated with managing complex health problems. One resident recounted his experience of sharing his life story, as his immobility progressed, which had unexpected benefits. He described talking with a student about *“…learning to walk on a walking stick,* [then a] *walking frame, and* [then a] *wheelchair…But as that happens over the years you take one* [step] *at a time and just get over it and get on with life*” (OP44_Resident). Sharing the story as a chronology had unexpected benefits of reinforcing coping self‐efficacy and adjustment to chronic illness. He suggested that, *“…to actually string the conversation together* [the story of this progression] *was the first time for me…I enjoyed it”* and that *“…I found it cathartic. It hit me emotionally and I had to stop a couple of times and that surprised me”* (OP44_Resident). This resident's wife also recounted his experience of teaching the student when she noted that he, *“…talked* [with students] *about the experience of living with MS* [Multiple Sclerosis]*…he was able to give a chronological story, a narrative, of what happened to him, and it's the first time he's really done that”* (OP49_Family member). She noted, *“…it was very therapeutically good for him*.*”* Further, this same family member reported that because she had worked for the MS Society she was, *“…able to give them* [the students] *lots of information about the clinical side of MS”* (OP49_Family member). Such comments highlight the deep level of engagement facilitated by the student placements and how this often fostered self‐reflection among residents and family members.

Residents were also willing to be experts by experience and took their responsibility as educators seriously. One argued, *“I think it's up to us* [residents] *to help* [the students] *through their course…they're trying to get on in life”* (OP43_Resident). This resident recounted how she taught students about her angina management, saying, *“I was able to tell them about angina…the levels of angina, how long each one lasts, and what it takes to take the worst pain away”* (OP43_Resident). There were a number of instances where residents’ health professional or educator background helped them to teach students and recover an element of their identity. One resident observed,

I was the clinical nurse consultant in charge of surgical floors in a hospital, so I am able to help. The nursing student was having trouble taking blood pressure and I suggested she bring the machine down [to me]…I went through it with her and gave her a few tips (OP02_Resident).

Reinforcing the above account, a staff member also noted that this resident, *“…thrives on training students… she enjoys getting their mind going with medical questions…She gets to use her brain and it makes her feel like she's the boss again”* (OP26_Staff). Staff members reported other perceived benefits associated with residents positioning themselves as teachers. For example, one recounted a conversation with a resident when she sought feedback on “…*what he liked most about having students”* (OP35_Staff). She noted that “*it heightens his enthusiasm …* [He said] *‘I'm giving them a little bit of information that they may not have known’*.*”* The nurse perceived that this resident, *“…really enjoys talking about his condition and the possibility of helping* [students] *have a better understanding”* (OP35_Staff). Opinions consistently aligned across all respondent groups, suggesting that residents obtained significant enjoyment in their interaction with students in ways that allowed them to act as mentors or teachers. This included imparting information about the lived experience of chronic disease, best approaches of treatment or care and supporting a future generation of health‐care professionals.

## 4 DISCUSSION

Older adults in RACFs are among the most high‐need populations within the health sector due to multiple morbidity, reduced mobility, chronic and progressive health conditions and sensory and cognitive impairments.^38^, ^39^ As evidence of the cohort's complex needs in the current study, six older adult participants died within six months of data collection, few had close relationships (i.e, most were widowed, divorced or never married, and reported limited family contact) and many experienced multiple chronic health conditions, including cognitive impairment and depressive symptoms. The quantitative data collected in this study reinforced the known vulnerabilities of aged care residents in other Australian reports^40^ that potentially have a negative influence on their QoL, including prevalent cognitive impairment and depression. Our findings suggest that the health status of older adults can influence their capacity to engage socially, and opportunities for social interaction in RACFs are limited. Students expand residents’ opportunities beyond the walls of the RACF, and they enable better health provision by adding human resources and increasing attention in a sector that often faces significant staffing pressures. Our findings, in combination with previous research,^13^ suggest that interactions with students as part of a TACF programme can foster important interaction and occupation for older adults when there are typically limited opportunities for meaningful engagement in RACF settings.

Social isolation and loneliness among community dwelling populations have been reported in numerous studies^41^, ^42^, ^43^ and are associated with reduced QoL and deleterious physical and mental health outcomes.^42^, ^44^ Despite the negative consequences and prevalence of isolation and loneliness among older adults, comparatively little research has investigated this phenomenon in RACFs.^15^ Some studies on isolation and loneliness in aged care settings suggest it may be more acute in such environments due to the compounding and negative effects of reduced social support and poor health.^6^, ^14^, ^15^ In support of this research, the current study showed residents experienced isolation and loneliness resulting from confinement to their rooms because of illness or mobility problems. This limited meaningful social interaction with staff and family members and influenced a lack of companionship with co‐residents who had cognitive or sensory issues.

In the context of social isolation and loneliness, student placements provided residents with socially supportive connections. Residents in this study appeared to be disconnected from social networks. There was an inability of family members and staff to commit quality time to residents, and residents experienced declines in health during transitions from home to institutional care. In this way, residents were suitable for a social support intervention, which could occur under similar circumstances, for example, when a social network is small, overburdened or unable to provide effective support and during times when individuals are experiencing major life transitions.^12^ Residents’ interactions with health students were highly valued by not only the residents, but their family members and RACF staff. For residents, students were seen to foster high‐quality social connection, facilitate access to new information about the outside world and provide companionship. Student placements moved beyond the pragmatic concerns for the provision of instrumental and informational support, but also fostered emotional support^12^ for frail older adults, which led to rapport building, depth of engagement and opportunities for resident empowerment. These outcomes are similar to those of psychosocial interventions, which are often effective for ameliorating isolation and loneliness among older adults,^41^ including participation in educational and social group activities and, in particular, interactions where older adults have opportunities to be active contributors.^4^, ^41^ The social processes that occurred during the health student placements may be similar to these kinds of psychosocial interventions, which in turn warrants further investigation.

Health student placements provided more than an avenue for supportive connections. They also gave older adults an opportunity to engage. Meaning making is central to psychological adjustment and human development as a process for responding to stressful or challenging situations through identity formation and reformation.^45^, ^46^ Meaning making is important in RACFs for the construction of a personal narrative that maintains coherence of individual identity or facilitates changes in identity during times of transition and age‐related losses.^47^, ^48^ In this research, older adults used their interaction with students to link back to their autobiographical memories as teachers, nurses and community leaders and reframe their current personal narrative from that of a vulnerable individual with high care needs to an experienced mentor who actively participates in educating future health professionals. This experience is similar to process inherent in reminiscence, which is defined as the process of thinking or telling someone about past experiences that are personally significant.^6^ Psychosocial interventions have adopted reminiscence approaches whereby autobiographical memories are retrieved alongside a reflective process that includes reframing negative experiences.^21^ A meta‐analysis^22^ showed that reminiscence has moderate positive effects on depressive symptoms, purpose in life and social integration. Our findings contribute to this research as the resident‐student interaction described above may extend part of the reminiscence approach whereby the process of passively recalling past memories can be extended by actively participating in forming new memories that are based on these past experiences (e.g, reframing a role as a former teacher into a current mentor).

Meaning making appeared to be created through a reciprocal engagement, which allowed for a renegotiation of social role and identity with implications for well‐being and QoL. This process reflects the underlying tenets of symbolic interactionism.^17^ The theory of Symbolic Interactionism provides explanation for how meaning and identity are created through significant social interactions.^18^ Blumer goes as far as to suggest that through the filter of cognition, human interaction with objects and people may indeed constitute our entire perception of reality.^18^ The world of aged care is arguably highly routinized, with care often shaped in terms of rules and rituals^49^ where the introduction of undergraduate health students provides an opportunity for vulnerable adults to reframe their reality and experience atypical, serendipitous social encounters. In their interactions with students, residents played the role of both the subject of health assessment and mentor to facilitate learning and positive interactions in aged care. This transaction was imbued with reciprocity to such an extent that many residents were actively engaged with teaching students about their health conditions or showing them the appropriate way to conduct a health assessment with a frail older person. Interactions with health students were different from those with RACF staff as they often included emotional and informational support. For example, when students shared poetry, brought flowers for residents and conversed about their lives, interests and course of study. In this interaction, students appeared to be opportunistically filling a gap in psychosocial care. The reciprocal relationship between students and aged care residents created the context for older adults to engage in valued social roles. In this research, the dual and reciprocal relationships appear to have a positive influence on older adults’ social connectedness; however, it also raises ethical issues with regard to managing professional boundaries^50^ when delivering psychological care. Future research could focus on implementation of psychology placements where students under supervision are able to deliver interventions (e.g, assessment and treatment for the presentations of depression and psychological adjustment to age‐related conditions). This may further contribute to understanding how the psychology discipline^51^ can enhance aged care placement experiences in RACFs.

**Figure 1:**
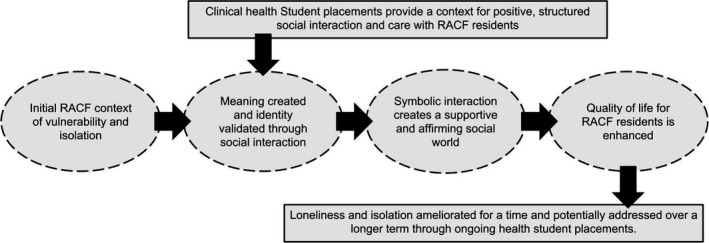
Depiction of the hypothesized effects of student‐resident interaction in a teaching aged care facility

While the aged care environment created the conditions for loneliness and isolation among residents, the structured student placements facilitated through the TACF programme created the conditions for addressing this isolation and thereby improving QoL. Such improvements were mediated through residents engaging in meaningful social interactions, which helped to affirm their personhood. Figure 1 depicts the hypothesized relationship between the themes that were identified in this analysis and the potential impacts on QoL.

## 5 LIMITATIONS

The results of this study are potentially limited by single‐site data collection and the influence of a long‐established student placement programme that had been underway for three years prior to the data collection. To verify and expand upon the current findings, confirmatory studies should be undertaken using a randomly allocated cohort of aged care residents in multiple TACFs. Larger cohorts of respondents will also allow for a more robust consideration of pre‐ and post‐student placement effects and experiences. The overarching challenge with research of this nature is that TACFs (facilities where student learning centres and clinical mentors are embedded within RACFs) remain a relatively novel educational context in Australia and similarly developed countries. Enhanced evaluation should proceed as more of these innovative facilities are developed in the years ahead. Furthermore, comparisons should also be made between the subjective and objective health and QoL outcomes reported at both long‐established and newly initiated TACF programmes to verify whether previous and incidental interactions with students over time have any effect on older adult QoL and quality of care. While the sample in this study was relatively small, participant engagement was high suggesting that residents were highly motivated to reflect upon their interactions with the students and support TACF programme evaluation. Moreover, the sample characteristics are broadly representative of the aged care clientele within Australia.^40^ The exploratory nature of this study and small sample indicates that the results, rather than generalizing to larger cohorts, should inform hypothesis generation as the number of global TACF programmes increases. In spite of the preliminary nature of the findings, the results suggest strong links between student placements and older adults’ QoL, which is mediated by meaningful social interactions.

## 6 CONCLUSION

In a large Australian RACF, isolation, loneliness and depression are prevalent among older adults. Structured and supervised health student placements create an opportunity to overcome isolation and loneliness by increasing socially supportive connections that engage older adults with a cohort of young learners. Through social interaction with students, residents become active participants in these transactions and have the opportunity to reframe themselves as mentors and educators, consolidating a meaningful identity. Interactions with health students support resident QoL in RACFs even in a context of vulnerability and high care needs, suggesting multiple benefits from the educational experience.

## CONFLICT OF INTEREST

None.
